# 

**Published:** 1880-02

**Authors:** 


					﻿Whole Number,
CCXXIII
FEBRUARY, 1880.
Number 7,
Volume XIX.
THE
BUFFALO
Medical and Surgical Journal.
EDITORS:
THOS. LOTHROI’, M.
P. W. VAN l’EVMA, M. D.,
A. R. DAVIDSON, M.
HERMAN MVNTER, M.
EtJCIEN HOWE, M. I>.
BUFFALO :
Published by the Medical Journal Association.
Read the Notice of this Journal among the Advertisements.
Entered at the Post-Office at Buffalo, N. Y., as Second-Class Mail Matter.
				

